# Nut and Peanut Butter Consumption and Mortality in the National Institutes of Health-AARP Diet and Health Study

**DOI:** 10.3390/nu11071508

**Published:** 2019-07-02

**Authors:** Vineeth Amba, Gwen Murphy, Arash Etemadi, ShaoMing Wang, Christian C. Abnet, Maryam Hashemian

**Affiliations:** 1 Metabolic Epidemiology Branch, Division of Cancer Epidemiology and Genetics, National Cancer Institute, National Institutes of Health, Rockville, MD 20850, USA; 2 The College of New Jersey, Ewing, NJ 08628, USA; 3 Digestive Oncology Research Center, Digestive Diseases Research Institute, Tehran University of Medical Sciences, 14117-13135 Tehran, Iran

**Keywords:** nut, peanut butter, NIH-AARP Diet and Health Study, mortality, cancer, cardiovascular disease, respiratory disease, chronic liver disease

## Abstract

Although previous studies have shown inverse associations between nut consumption and mortality, the associations between nut consumption and less common causes of mortality have not been investigated. Additionally, about 50% of peanut consumption in the US is through peanut butter but the association between peanut butter consumption and mortality has not been thoroughly evaluated. The National Institutes of Health-AARP (NIH-AARP) Diet and Health Study recruited 566,398 individuals aged 50–71 at baseline in 1995–1996. A food-frequency questionnaire was used to evaluate nut and peanut butter consumption. Cox proportional hazard models were used to estimate hazard ratios and 95% confidence intervals for mortality using the non-consumers as reference groups and three categories of consumption. After excluding subjects with chronic diseases at baseline, there were 64,464 deaths with a median follow-up time of 15.5 years. We observed a significant inverse association between nut consumption and overall mortality (HR _C4 vs C1_ = 0.78, 95% CI = 0.76, 0.81, *p* ≤ 0.001). Nut consumption was significantly associated with reduced risk of cancer, cardiovascular, respiratory, infectious, renal and liver disease mortality but not with diabetes or Alzheimer’s disease mortality. We observed no significant associations between peanut butter consumption and all-cause (HR _C4 vs C1_ = 1.00, 95% CI = 0.98, 1.04, *p* = 0.001) and cause-specific mortality. In a middle-aged US population, nut intake was inversely associated with all-cause mortality and certain types of cause-specific mortality. However, peanut butter consumption was not associated with differential mortality.

## 1. Introduction

Previous studies have found significant inverse associations between nut consumption and overall mortality [1,2,3,4,5,6,7,8,9,10,11,12,13,14] as well as between nut consumption and cardiovascular (CVD) disease [6,8,9,12,15] and multiple cancers including pancreatic [16], colorectal [17], esophageal squamous cell carcinoma [18] and gastric noncardiac adenocarcinoma [19,20]. A past review study on nut intake and mortality reported all-cause and common causes of mortality such as cardiovascular and cancer mortality [5]. But, the association between nut consumption and less common causes of mortality has not been investigated. Also, although 50% of peanut consumption in the United States is through peanut butter [21], few studies have examined health outcomes related to the popular food. The only study on peanut butter and mortality in Netherland [13] has found mainly insignificant conclusions in a case-cohort study with 8823 deaths and 3202 sub cohort members.

In this study, we investigated the association between nut and peanut butter consumption with mortality using data from the National Institutes of Health-AARP (NIH-AARP) Diet and Health Study (ClinicalTrial.gov # NCT00340015), which had more than 128,000 deaths. This is comparable to a recent metanalysis on nut consumption and mortality [8]. Due to the large size of the study, we were able to exclude participants with self-reported chronic diseases at baseline as they could have altered diets and conduct subgroup analysis by key variables such as sex, education and body-mass index (BMI). The study contains extensive information on contextual variables, as well as data on less commonly reported forms of mortality. Our results can help provide further insight on the health outcomes associated with nut and peanut butter consumption.

## 2. Materials and Methods

The NIH-AARP prospective cohort study consists of a group of 566,398 people from six different states (California, Florida, Louisiana, New Jersey, North Carolina, Pennsylvania) and two cities, (Atlanta, Georgia and Detroit, Michigan) [22]. The cohort consists of 227,021 women and 340,148 men. Originally in 1995–1996, 3.5 million members of the AARP (50–71 years old), were sent a questionnaire that asked for information about typical diet, demographic characteristics and health-related behaviors [22]. 617,119 individuals responded to the questionnaire at a 17.6 % response rate but 50,721 were excluded for reasons including recording errors, requests to be removed from the study and incomplete responses [22]. In this analysis, we excluded those who self-reported a history of cancer, diabetes, emphysema, end-stage renal disease, heart disease and stroke in all analyses to account for observations that may have been affected by changes in dietary patterns. For the nut analysis, those who did not answer information about nut consumption frequency and nut intake portion size were excluded. Similarly, in the peanut butter analysis, those who did not answer information about peanut butter consumption frequency and peanut butter intake portion size were also removed. The final size of the cohort included in the nut portion of the study was 374,101 participants and the peanut butter portion contained 380,351 participants. The National Cancer Institute (NCI) and the Westat, Incorporated. institutional review boards approved the NIH-AARP Diet and Health study [23].

### 2.1. Dietary Assessment

A baseline food-frequency questionnaire (FFQ) that was mailed to the members of the cohort to obtain dietary information. The FFQ inquired about the consumption of 124 different items over the preceding 12 months [22]. To validate the FFQ further, a subset of respondents was given two non-consecutive 24-hour recall interviews [22]. Nut intake was represented as “peanuts, walnuts, seeds or other nuts” on the FFQ and was divided into ten categories of frequencies, including “never,” “1–6 times per year,” “7–11 times per year,” “1 time per month,” “2–3 times per month,” “1–2 times per week,” “3–4 times per week,” “5–6 times per week,” “1 time per day” and “2+ times per day.” This information was supplemented by three portion size categories, including “less than 1/4 cup,” “1/4 to 1/2 cups” and “more than 1/2 cup.” Peanut butter consumption was shown as “peanut butter or other nut butter” and was divided into the same frequency categories. However, the three portion size categories were “less than one tablespoon,” “one to two tablespoons” and “more than two tablespoons.” Nut and peanut butter intake (in grams) were calculated by utilizing information on portion size and frequency of intake. The mass of a quarter cup of nuts is 32.75 grams and the mass of a tablespoon of peanut butter is 16 grams [19]. Energy intake was computed using the nutrient database from the 1994–1996 Continuous Survey of Food Intake by Individuals of the US Department of Agriculture [24]. 

### 2.2. Cause of Death Ascertainment

Death, from any cause, was the main endpoint for the study. The actual vital status was confirmed by the linkage to the Death Master File, which contains death information reported to the Social Security Administration of the United States [25]. Details regarding the NIH-AARP Diet and Health Study cohort design have been previously explained [22,23].

### 2.3. Statistical Analysis

We tabulated nut and peanut butter consumption in relation to potential risk factors of mortality. Cox proportional hazard models were used to estimate hazard ratios (HRs) and 95% confidence intervals (95% CIs) for total and cause-specific mortality. The proportional hazards assumption was not violated using the Schoenfeld residuals test. The scale used for time was person-years and was recorded from the point of return of the original FFQ to the point of death for any reason, loss to follow-up, end of follow-up period (12/31/2011) or movement out of registry area, whichever came first. Using age as the underlying time measure did not significantly impact the results. The nutrition density model was used for energy adjustment and the categories of nut and peanut butter consumption were based on grams of intake per 1000 kilocalories [26], with category one consisting of individuals who did not consume nuts. Energy intake was still accounted for in the multivariate nutrient density model [26]. 

Two adjusted models were used, one adjusted for only sex and age and one adjusted for multiple variables. For the multivariable-adjusted models, we investigated multiple confounding variables. These included age (continuous), sex, body-mass index (BMI) (<25 kg/m^2^, ≥25 to <30 kg/m^2^, ≥30 kg/m^2^), race (non-Hispanic white; non-Hispanic black; Hispanic, Asian, Pacific Islander, American Indian/ Alaskan Native), smoking status and dose (never, former ≤20 cigarettes/ day, former >20 cigarettes/day, current ≤20 cigarettes/day, current >20 cigarettes/day), alcohol consumption (grams/day), education level (1–11 years of schooling, 12 years of schooling or completed high school, post-high school or some college education, college and postgraduate education), physical activity (never, rarely, 1–3 times/month, 1–2 times/week, 3–4 times/week, ≥5 times/week), white meat intake (grams/day), red meat intake (grams/day), vegetable intake (grams/day), fruit intake (grams/day), whole grain intake (grams/day), calorie intake (kilocalories/day) and use of vitamins (yes or no) [27]. Category one served as the reference and consisted of individuals who had not consumed nuts or peanut butter. Categories two, three and four represented tertiles of consumption in grams. These analyses were conducted for all-cause mortality, cancer mortality, CVD disease mortality, respiratory disease mortality, diabetes mortality, mortality from infectious causes, Alzheimer’s Disease mortality, chronic liver disease mortality, nephritis/ nephrotic syndrome/ nephrosis mortality, accident/ suicide/ homicide mortality and other/ unknown cause mortality. Then, median values from each category were taken to assess the linear trend across categories of consumption. Continuous scale calculations were also carried out using 2.15 grams of increased nut consumption and 1.7 grams of increased peanut butter consumption. These values were obtained by subtracting the 25^th^ percentile of consumption in grams from the 75^th^ percentile of consumption.

In an additional analysis, frequency data for nut and peanut butter intake was used to create two categories, one consisting of those who ate nuts/ peanut butter and the other category consisting of those who never ate nuts/ peanut butter or only ate nuts/peanut butter one to six times per year. By using the likelihood ratio test, interactions between the two categories were analyzed using sex, age, education, smoking status and BMI. The interaction likelihood ratio test was significant when *p* < 0.05. In a lag analysis we excluded participants with less than two years of follow-up to observe how reverse causation could have affected data. All analysis was carried out through the STATA software (version 15; STATA).

## 3. Results

### 3.1. Baseline Factors of Nut and Peanut Butter Consumers

The mean (±SD) of nut intake was 2.9 ± 8.3 grams/day and peanut butter intake was 2.9 ± 7.1. The median age of the overall cohort was 61.9 years and males comprised approximately 56.4% of the group. Additionally, 41.3% of the cohort had either completed college and/or had postgraduate education. 

Baseline traits of the 374,101 individuals included are shown in Table 1. Those who consumed greater quantities of nuts also consumed greater quantities of alcohol, white meat, red meat, vegetables, fruits and whole grains. Generally, nut consumers were also more likely to be younger, men, consist of former smokers rather than current smokers, exercise more often, use vitamins and reported their current health condition as excellent. The table presents characteristics of the 380,351 participants included in the peanut butter analysis. Those who consumed greater quantities of peanut butter consumed lesser quantities of alcohol. Mainly, peanut butter consumers were more likely to have a BMI greater than 30 kg/m^2^, be Non-Hispanic White, smoke at a greater rate, exercise 3–4 times per week and consume more red meat and whole grains. They were also less likely to self-report their health condition as excellent.

### 3.2. Nut Consumption and Mortality

In the median follow-up time of 15.5 years (5,798,566 person-years), there were 64,464 total deaths. Table 2 demonstrated a significant inverse association between nut consumption and overall mortality (HR _C4 vs C1_ = 0.78, 95% CI = 0.76, 0.81, *p* ≤ 0.001). Nut consumption was significantly associated with decreased risk of cancer, cardiovascular, respiratory, infectious, renal and liver disease mortality but not with diabetes and Alzheimer’s disease mortality. Other than a 5% decreased risk of developing respiratory disease, other categories of mortality did not show a significant association for every 2.15 grams increase in nut consumption.

### 3.3. Peanut Butter Consumption and Mortality

There was a total of 65,850 deaths with a median follow-up time of 15.5 years or 5,895,441 person-years. There was no significant association between peanut butter intake and all-cause (HR _C4 vs C1_ = 1.00, 95% CI = 0.98, 1.04, *p* = 0.001) and cause-specific mortality (Table 3). However, *p* trends were significant for overall, cardiovascular and respiratory disease (0.001, 0.02 and 0.05, respectively). We did not find any significant association for every 1.7 grams of increase in peanut butter consumed per 1000 kcal/d. 

### 3.4. Subgroup and Sensitivity Analyses

In the subgroup analysis, participants who ate nuts were compared against a group that contained those who never ate nuts and those who only ate nuts one to six times per year. Stratifications by sex, age, education, smoking status, BMI and health condition were executed. The significant inverse relationship between nut intake and mortality was noticed in all subgroups with no significant interaction (Table 4). Borderline significant interaction was seen for peanut butter consumption and BMI but differences in risk estimated between the strata were modest.

After excluding the first two years of follow-up for the nut intake cohort, the association was similar to the overall estimate (HR _C4 vs C1_ = 0.74, 95% CI = 0.64, 0.86, *p* ≤ 0.001). This sensitivity analysis did not alter the null association between peanut butter intake and mortality (HR _C4 vs C1_ = 1.02, 95% CI = 0.90, 1.15, *p* = 0.37).

## 4. Discussion

In this large prospective cohort study, there was a significant inverse association between nut intake and all-cause and cause-specific mortality, including cancer, CVD, respiratory disease, infectious causes, chronic liver disease and renal disease. However, no significant associations between peanut butter consumption and all-cause and cause-specific mortality were observed. 

There were similar findings in other nut intake studies with a smaller number of death counts. In a study by Bao et al., there were significant inverse associations between nut intake and all-cause, cancer, CVD and respiratory disease mortality [1,2]. Other papers found similar associations in a variety of different populations [3,4,5,6,7,8,9,10,11,12,13,15], even with different socioeconomic statuses and lifestyles [2]. 

The relatively large reduction in risk in the first category of nut consumption may indicate that there is little additional benefit for nut consumption at higher levels of consumption. Also, some of the remaining effect could be due to residual confounding and represent lifestyle factors associated with being a nut consumer, rather than the effects of the nuts themselves. However, we comprehensively assessed potential confounders using the data available in the NIH-AARP questionnaire that have been associated with mortality and assessed whether these factors confounded the association between nut intake and mortality. Nevertheless, several hypotheses may explain a potential beneficial association between nut intake and mortality. Nuts contain dietary fiber which may play a part in reducing risk of heart disease, colorectal cancers [28] and other causes of death. Additionally, nuts contain large amounts of monounsaturated fatty acids [28]. It was found that walnut consumption could lower levels of LDL-cholesterol and advantageously change lipoprotein profiles in men [29,30]. Tocopherols and phytochemicals in nuts play a role as antioxidants and regulators of cancerous cell growth [31,32,33,34]. Other elements of nuts, such as flavonoids and resveratrol, also can promote apoptosis and reduce carcinogenesis [28,35] and decrease cardiovascular disease through several mechanisms [36]. 

Interestingly, a robust significant inverse association between nut intake and respiratory disease mortality was present. As noted above for total mortality, this could be explained by residual confounding by smoking status or the antioxidants obtained from nuts, as they can prevent damage to the respiratory tract by oxidative stress [37]. Additionally, tocopherols in nuts can contribute to reducing lipid degradation, which can lead to unhealthy lung conditions [37]. We also cannot rule out reverse causation, because patients suffering from chronic obstructive pulmonary disease (COPD) may have had different diets due to difficulty swallowing certain foods which could have impacted the associations we observed [38].

Although nut butter is defined as a food that contains 90% nut product [39], we did not observe any significant associations between peanut butter consumption and mortality. There are several explanations for this observation: First, peanut butter consumers had several lifestyle factors that are known to adversely affect health including being more likely to consume red meat, to currently smoke cigarettes and were less likely to exercise. Second, the processing of peanut butter may affect the beneficial effects of nuts. Third, nut consumption in this population is largely from nuts other than peanuts whereas a large proportion of nut butter is peanut butter. Peanuts may not individually cause some of the health benefits that were observed for nut consumption. Different types of nuts contain different amount of fatty acids, minerals and vitamins. All of these nutrients have been proposed to be associated with mortality including cardiovascular and cancer mortality in experimental and epidemiological studies [40,41,42,43,44,45]. Our results are consistent with a study by Van den Brandt et al., which found an insignificant association between peanut butter consumption and mortality [13]. Sui et al. also showed inverse association between tree nut consumption and hepatocellular carcinoma but no association between peanut or peanut butter and hepatocellular carcinoma [46].

Our study has many strengths. Our analysis had a large sample size and we excluded subjects with self-reported health conditions at baseline. There was also a sizable number of deaths during follow-up and accurate death ascertainment for a variety of different diseases. This allowed us to explore a greater variety of health outcomes in comparison to previous papers in the subject area. There are important limitations to note as well. We collected data on all nut consumption without accounting for specific nut characteristics that could influence health outcomes. These include intake of raw, roasted, salted, spiced and mixed nuts. Additionally, nut and peanut butter consumption were self-reported, so measurement error could have occurred. We also used a single dietary report which cannot account for changes in dietary intake of foods during the study period [47]. Our population was also between the ages of 50 and 71 during the study, more likely to be Non-Hispanic White and was more educated then the general US population, which may limit the generalizability of our findings. Finally, this is an observational study, so we cannot assume cause and effect associations between nut/peanut butter consumption and mortality exist.

## 5. Conclusions

We found a significant inverse association between nut consumption and mortality, but overall, no association between peanut butter consumption and mortality. It could be useful to conduct future studies that ask participants about different types of nuts (walnuts, almonds, etc.) and how the nuts they consume are prepared (roasted, salted, spiced, etc.). Additionally, as this is only the second prospective cohort study conducted on peanut butter consumption and mortality, further observational studies are warranted to learn more about the health outcomes associated with this popular food. Researching the biological mechanisms in nuts should also continue to be a priority.

## Figures and Tables

**Table 1:** Baseline characteristics of participants by categories of dietary nut and peanut butter intake in the NIH-AARP Diet and Health Study ^1^.

	Nut Intake Categories (*n* = 374, 101)				Peanut Butter Categories (*n* = 380, 351)			
	C1 (*n* = 32, 443)	C2 (*n* = 110, 614)	C3 (*n* = 113, 832)	C4 (*n* = 117, 212)	C1 (*n* = 79, 464)	C2 (*n* = 103, 965)	C3 (*n* = 98, 574)	C4 (*n* = 98, 348)
Nut or peanut butter intake, g/1000 kcal	0	0.11 (0.05–0.17)	0.51 (0.36–0.68)	2.20 (1.37–4.14)	0	0.14 (0.08–0.21)	0.63 (0.45–0.89)	3.00 (1.99–5.78)
Age at baseline, y	62.9 (57.9–66.8)	62.0 (57.2–66.2)	61.6 (56.9–65.9)	61.9 (57.2–66.1)	62.1 (57.3–66.3)	61.6 (56.8–66.0)	61.9 (57.2–66.1)	62.3 (57.6–66.4)
Male sex, %	50.0	51.0	57.4	62.3	57.9	50.5	53.9	63.6
BMI, kg/m^2^, %								
<25	39.8	39.5	35.8	37.4	41.5	37.7	36.4	36.2
≥25	39.4	41.2	43.6	43.7	41.9	42.2	42.5	43.4
≥30	20.8	19.3	20.7	18.9	16.6	20.1	21.1	20.4
Race, %								
Non-Hispanic White	92.3	92.6	93.2	92.6	90.8	91.1	93.5	95.1
Non-Hispanic Black	4.0	3.5	3.5	3.9	3.6	4.7	3.7	2.9
Other	3.7	3.9	3.3	3.5	5.6	3.7	2.8	2.1
Smoking Status, %								
Never	37.3	40.4	39.1	38.3	38.5	40.2	39.6	38.2
Former								
≤20 cigarettes/d	26.8	28.5	29.2	30.0	29.3	29.2	29.0	28.7
>20 cigarettes/d	19.3	18.0	19.5	20.4	20.9	18.6	18.5	19.5
Current								
≤20 cigarettes/d	11.1	8.8	8.0	7.3	7.7	8.0	8.6	8.7
>20 cigarettes/d	5.7	4.3	4.1	4.0	3.6	4.0	4.3	4.9
Alcohol, g/d	0.9 (0–7.0)	1.7 (0.2–10.9)	2.4 (0.4–12.4)	3.1 (0.5–14.8)	2.7 (0.3–15.2)	2.2 (0.4–13.4)	2.0 (0.3–11.0)	1.8 (0.2–10.4)
Education Level, %								
1–11 years of schooling	37.0	27.6	24.0	20.2	24.1	25.1	25.5	25.3
12 years/high school	9.9	10.7	10.1	9.2	9.1	10.1	10.1	10.4
High School/some college	22.7	24.1	23.8	23.7	23.2	24.0	23.9	23.7
College/postgraduate	30.5	37.6	42.2	46.9	43.6	40.8	40.5	40.6
Physical activity, %								
Never	8.1	4.0	3.2	2.8	4.8	3.6	3.3	3.4
Rarely	16.3	13.6	12.6	11.3	13.1	13.2	12.8	12.2
1–3 times/mo	12.8	14.4	14.5	13.6	12.7	14.7	14.8	13.8
1–2 times/wk	18.8	22.3	23.3	23.0	20.7	22.5	23.4	23.0
3–4 times/wk	23.7	26.9	27.6	28.8	26.8	27.1	27.7	28.0
≥5 times/wk	20.3	18.8	18.8	20.5	21.9	18.9	18.1	19.7
White meat intake, g/d	40.7 (20.3–73.4)	43.2 (24.3–73.2)	45.9 (26.3–76.5)	48.2 (27.7–79.8)	46.0 (24.7–79.2)	45.8 (25.3–78.4)	44.6 (26.0–73.8)	44.9 (25.7–74.6)
Red meat intake, g/d	39.5 (18.6–74.4)	46.8 (25.3–79.5)	52.3 (29.1–88.0)	55.3 (29.8–92.7)	41.8 (19.7–77.1)	48.5 (25.9–84.6)	52.5 (30.1–85.0)	56.6 (31.7–94.1)
Vegetable intake, g/d	231.5 (141.1–362.9)	245.1 (161.3–360.8)	250.0 (166.0–366.9)	266.5 (177.1–389.6)	252.6 (161.0–382.7)	256.9 (167.6–383.7)	246.7 (165.5–355.8)	251.6 (166.2–369.2)
Fruit intake, g/d	275.7 (134.7–471.4)	294.2 (161.1–474.9)	289.1 (158.6–461.1)	298.9 (165.7–477.2)	298.5 (156.9–495.9)	301.9 (165.2–493.0)	287.4 (158.4–452.1)	284.1 (156.2–449.9)
Whole grain intake, g/d	9.1 (0–28.5)	12.4 (1.3–33.8)	12.6 (1.7–31.8)	12.8 (2.0–32.6)	9.8 (0.5–30.8)	11.3 (1.5–31.8)	12.4 (1.7–30.8)	15.9 (1.7–35.3)
Calories, kcal/d	1541 (1138–2076)	1621 (1278–2108)	1664 (1250–2228)	1823 (1371–2320)	1580 (1189–2080)	1703 (1244–2272)	1645 (1319–2046)	1830 (1344–2409)
Uses vitamins, %	53.0	56.1	56.4	57.5	54.9	56.9	56.8	56.1
Self-reported health condition, %								
Excellent	19.1	20.8	21.4	23.2	24.3	21.5	20.2	20.8
Very Good	35.8	40.3	41.2	41.7	39.8	40.2	41.1	41.0
Good	35.1	32.4	31.7	29.9	29.7	32.0	32.6	32.0
Fair/ Poor	10.1	6.5	5.8	5.2	6.3	6.3	6.2	6.2

NOTE: Certain percentage categories do not equal 100 due to rounding or missing data. ^1^ Values are presented as either median (IQR) or percentages. Intake density is based on gram per 1000 kcal. C, category. All variables were associated with nut and peanut butter consumption, *p* < 0.001.

**Table 2:** Crude, sex and age-adjusted and multivariable-adjusted hazard ratios of types of death, by categories of nut intake (*n* = 374,101) ^1^.

	Nut Intake Categories					
	C1	C2	C3	C4	*p*-Value for Trend	Continuous ^3^
Person-years, *n*	459,656	1,610,365	1,665,839	1,717,243		
Nut median intake (IQR), g/1000 kcal	0	0.11 (0.05–0.17)	0.51 (0.36–0.68)	2.20 (1.37–4.14)		
All-Cause						
Cases of death, *n*	7536	19,351	18,602	18,975		
Crude HR (95% CI)	1.00	0.73 (0.71–0.75)	0.67 (0.65–0.69)	0.67 (0.65–0.68)	<0.001	0.99 (0.98–0.99)
Sex and Age-Adjusted HR (95% CI)	1.00	0.76 (0.74–0.78)	0.71 (0.69–0.72)	0.67 (0.66–0.69)	<0.001	0.98 (0.97–0.98)
Multivariable-Adjusted HR (95% CI) ^2^	1.00	0.84 (0.82–0.87)	0.80 (0.77–0.82)	0.78 (0.76–0.81)	<0.001	0.99 (0.98–1.00)
Cancer						
Cases of death, *n*	2763	7957	7823	8142		
Crude HR (95% CI)	1.00	0.82 (0.78–0.85)	0.77 (0.74–0.81)	0.78 (0.75–0.82)	<0.001	0.99 (0.98–1.00)
Sex and Age-Adjusted HR (95% CI)	1.00	0.85 (0.81–0.88)	0.80 (0.77–0.84)	0.79 (0.75–0.82)	<0.001	0.99 (0.98–0.99)
Multivariable-Adjusted HR (95% CI) ^2^	1.00	0.91 (0.87–0.95)	0.88 (0.83–0.92)	0.88 (0.84–0.92)	0.003	0.99 (0.98–1.00)
Cardiovascular Disease						
Cases of death, *n*	2190	5162	4939	4971		
Crude HR (95% CI)	1.00	0.67 (0.63–0.70)	0.61 (0.58–0.65)	0.60 (0.57–0.63)	<0.001	0.98 (0.97–0.99)
Sex and Age-Adjusted HR (95% CI)	1.00	0.70 (0.67–0.74)	0.65 (0.62–0.68)	0.61 (0.58–0.64)	<0.001	0.97 (0.96–0.98)
Multivariable-Adjusted HR (95% CI) ^2^	1.00	0.78 (0.73–0.82)	0.72 (0.68–0.76)	0.70 (0.66–0.74)	<0.001	0.99 (0.98–1.00)
Respiratory Disease						
Cases of death, *n*	633	1290	1193	1072		
Crude HR (95% CI)	1.00	0.57 (0.52–0.63)	0.51 (0.46–0.56)	0.44 (0.40–0.49)	<0.001	0.93 (0.91–0.95)
Sex and Age-Adjusted HR (95% CI)	1.00	0.61 (0.56–0.67)	0.56 (0.51–0.62)	0.47 (0.43–0.52)	<0.001	0.93 (0.90–0.95)
Multivariable-Adjusted HR (95% CI) ^2^	1.00	0.76 (0.68–0.85)	0.73 (0.66–0.81)	0.64 (0.57–0.72)	< 0.001	0.95 (0.92–0.97)
Diabetes						
Cases of death, *n*	71	197	193	200		
Crude HR (95% CI)	1.00	0.78 (0.59–1.02)	0.73 (0.56–0.96)	0.74 (0.56–0.97)	0.32	0.96 (0.90–1.02)
Sex and Age-Adjusted HR (95% CI)	1.00	0.81 (0.61–1.06)	0.75 (0.57–0.98)	0.73 (0.56–0.96)	0.15	0.95 (0.89–1.01)
Multivariable-Adjusted HR (95% CI) ^2^	1.00	1.01 (0.73–1.38)	0.92 (0.67–1.27)	0.95 (0.69–1.31)	0.74	0.96(0.90–1.02)
Infectious Causes						
Cases of death, *n*	181	419	370	372		
Crude HR (95% CI)	1.00	0.65 (0.55–0.78)	0.56 (0.47–0.66)	0.54 (0.45–0.65)	<0.001	0.96 (0.92–1.00)
Sex and Age-Adjusted HR (95% CI)	1.00	0.69 (0.58–0.82)	0.59 (0.49–0.70)	0.56 (0.47–0.67)	<0.001	0.96 (0.92–1.00)
Multivariable-Adjusted HR (95% CI) ^2^	1.00	0.77 (0.64–0.94)	0.73 (0.60–0.89)	0.72 (0.59–0.88)	<0.001	0.98 (0.94–1.02)
Alzheimer’s Disease						
Cases of death, *n*	146	462	364	413		
Crude HR (95% CI)	1.00	0.88 (0.73–1.06)	0.67 (0.55–0.81)	0.74 (0.61–0.89)	0.019	1.00 (0.97–1.03)
Sex and Age-Adjusted HR (95% CI)	1.00	0.97 (0.80–1.16)	0.76 (0.63–0.92)	0.81 (0.67–0.97)	0.029	1.00 (0.96–1.03)
Multivariable-Adjusted HR (95% CI) ^2^	1.00	1.03 (0.83–1.27)	0.85 (0.68–1.05)	0.86 (0.69–1.07)	0.062	0.99 (0.96–1.03)
Chronic Liver Disease						
Cases of death, *n*	89	209	202	157		
Crude HR (95% CI)	1.00	0.67 (0.52–0.85)	0.62 (0.49–0.80)	0.47 (0.36–0.61)	<0.001	0.96 (0.90–1.02)
Sex and Age-Adjusted HR (95% CI)	1.00	0.67 (0.53–0.87)	0.61 (0.48–0.79)	0.44 (0.34–0.58)	<0.001	0.95 (0.89–1.01)
Multivariable-Adjusted HR (95% CI) ^2^	1.00	0.79 (0.60–1.04)	0.75 (0.57–1.00)	0.62 (0.46–0.83)	0.006	0.99 (0.94–1.05)
Nephritis, Nephrotic Syndrome and Nephrosis						
Cases of death, *n*	95	218	196	194		
Crude HR (95% CI)	1.00	0.64 (0.51–0.82)	0.56 (0.44–0.71)	0.53 (0.42–0.68)	0.004	0.99 (0.94–1.04)
Sex and Age-Adjusted HR (95% CI)	1.00	0.68 (0.53–0.87)	0.59 (0.46–0.76)	0.54 (0.42–0.69)	0.001	0.98 (0.93–1.03)
Multivariable-Adjusted HR (95% CI) ^2^	1.00	0.74 (0.56–0.97)	0.68 (0.51–0.89)	0.63 (0.48–0.84)	0.037	1.01 (0.96–1.06)
Accident, Suicide or Homicide						
Cases of death, *n*	234	639	666	669		
Crude HR (95% CI)	1.00	0.77 (0.67–0.90)	0.78 (0.67–0.90)	0.76 (0.65–0.88)	0.113	1.03 (1.00–1.05)
Sex and Age-Adjusted HR (95% CI)	1.00	0.79 (0.68–0.92)	0.78 (0.67–0.90)	0.72 (0.62–0.84)	0.005	1.02(0.99–1.04)
Multivariable-Adjusted HR (95% CI) ^2^	1.00	0.84 (0.71–0.99)	0.84 (0.71–0.99)	0.81 (0.69–0.96)	0.202	1.03 (1.00–1.05)
Other/ Unknown Causes of Death						
Cases of death, *n*	1049	2577	2437	2586		
Crude HR (95% CI)	1.00	0.69 (0.64–0.74)	0.63 (0.59–0.68)	0.65 (0.60–0.70)	<0.001	0.99 (0.97–1.00)
Sex and Age-Adjusted HR (95% CI)	1.00	0.73 (0.68–0.78)	0.67 (0.62–0.72)	0.67 (0.62–0.72)	<0.001	0.98 (0.97–0.99)
Multivariable-Adjusted HR (95% CI) ^2^	1.00	0.73 (0.68–0.78)	0.67 (0.62–0.72)	0.67 (0.62–0.72)	0.005	0.99 (0.98–1.01)

^1^ Intake density is based on gram per 1000 kcal. C, Category. IQR, Inter Quartile Range; ^2^ Multivariable models were adjusted for age (years), sex (male or female), BMI (<25, ≥25 to <30, ≥30), level of education (1–11 years of schooling, 12 years/high school, high school/college, college/ postgraduate), race (Non-Hispanic White, Non-Hispanic Black, Other), self-reported health condition (excellent, very good, good, fair/ poor), smoking status (never, former: ≤20 cigarettes/d, former: >20 cigarettes/d, current: ≤20 cigarettes/d, current: >20 cigarettes/d), total energy consumption (in kilocalories per day), alcohol consumption (grams per day), vitamin consumption (yes or no), physical activity (never, rarely, 1–3 times/month, 1–2 times/week, 3–4 times/week, ≥5 times/week) and food groups including white meat (grams per day), red meat (grams per day), whole grain (grams per day), vegetable (grams per day) and fruit (grams per day). ^3^ Continuous for every 2.15 grams of increased nut consumption.

**Table 3:** Sex and age-adjusted and multivariable-adjusted hazard ratios of types of death, by categories of peanut butter intake (*n* = 380,351) ^1^.

	Peanut Butter Intake Categories					
	C1	C2	C3	C4	*p*-Value for Trend	Continuous ^3^
Person-years, *n*	79,464	1,521,858	1,436,899	1,425,829		
Peanut butter median intake (IQR), g/1000 kcal/day	0	0.14 (0.08–0.21)	0.63 (0.45–0.89)	3.00 (1.99–5.78)		
All-Cause						
Cases of death, *n*	13,887	16,820	16,839	18,304		
Crude HR (95% CI)	1.00	0.92 (0.90–0.94)	0.98 (0.95–1.00)	1.07 (1.05–1.10)	<0.001	1.01 (1.00–1.01)
Sex and Age-Adjusted HR (95% CI)	1.00	0.97 (0.95–0.99)	1.00 (0.98–1.02)	1.04 (1.02–1.06)	<0.001	1.01 (1.00–1.01)
Multivariable-Adjusted HR (95% CI) ^2^	1.00	0.95 (0.92–0.97)	0.97 (0.95–1.00)	1.00 (0.98–1.04)	0.001	1.00 (1.00–1.01)
Cancer						
Cases of death, *n*	5704	7091	6913	7414		
Crude HR (95% CI)	1.00	0.94 (0.91–0.98)	0.98 (0.94–1.01)	1.06 (1.02–1.09)	<0.001	1.01 (1.00–1.01)
Sex and Age-Adjusted HR (95% CI)	1.00	0.99 (0.96–1.02)	1.00 (0.96–1.03)	1.03 (1.00–1.07)	0.012	1.01 (1.00–1.01)
Multivariable-Adjusted HR (95% CI) ^2^	1.00	0.97 (0.93–1.00)	0.96 (0.92–1.00)	0.98 (0.95–1.02)	0.646	1.00 (0.99–1.00)
Cardiovascular Disease						
Cases of death, *n*	3797	4382	4546	4959		
Crude HR (95% CI)	1.00	0.88 (0.84–0.91)	0.96 (0.92–1.01)	1.06 (1.02–1.11)	<0.001	1.01 (1.01–1.02)
Sex and Age-Adjusted HR (95% CI)	1.00	0.94 (0.90–0.98)	0.99 (0.95–1.04)	1.02 (0.98–1.07)	0.003	1.01 (1.00–1.01)
Multivariable-Adjusted HR (95% CI) ^2^	1.00	0.90 (0.86–0.94)	0.96 (0.91–1.01)	0.99 (0.95–1.04)	0.021	1.01 (1.00–1.01)
Respiratory Disease						
Cases of death, *n*	838	1065	1141	1243		
Crude HR (95% CI)	1.00	0.96 (0.88–1.05)	1.10 (1.00–1.20)	1.21 (1.11–1.32)	<0.001	1.01 (1.01–1.02)
Sex and Age-Adjusted HR (95% CI)	1.00	1.00 (0.92–1.10)	1.11 (1.02–1.22)	1.19 (1.09–1.30)	<0.001	1.01 (1.01–1.02)
Multivariable-Adjusted HR (95% CI) ^2^	1.00	1.00 (0.90–1.11)	1.09 (0.98–1.20)	1.10 (1.00–1.21)	0.045	1.00 (0.99–1.01)
Diabetes						
Cases of death, *n*	124	170	200	192		
Crude HR (95% CI)	1.00	1.04 (0.82–1.31)	1.29 (1.03–1.62)	1.26 (1.01–1.58)	0.058	1.01 (1.00–1.03)
Sex and Age-Adjusted HR (95% CI)	1.00	1.10 (0.87–1.39)	1.33 (1.06–1.66)	1.22 (0.97–1.53)	0.250	1.01 (0.99–1.02)
Multivariable-Adjusted HR (95% CI) ^2^	1.00	1.00 (0.76–1.30)	1.26 (0.98–1.62)	1.12 (0.87–1.46)	0.498	1.00 (0.98–1.02)
Infectious Causes						
Cases of death, *n*	280	362	335	402		
Crude HR (95% CI)	1.00	0.98 (0.84–1.15)	0.96 (0.82–1.13)	1.17 (1.00–1.36)	0.005	1.02 (1.01–1.03)
Sex and Age-Adjusted HR (95% CI)	1.00	1.03 (0.88–1.20)	0.98 (0.84–1.15)	1.14 (0.98–1.33)	0.037	1.01 (1.00–1.02)
Multivariable-Adjusted HR (95% CI) ^2^	1.00	0.96 (0.81–1.15)	0.96 (0.81–1.15)	1.10 (0.92–1.30)	0.092	1.01 (0.99–1.02)
Alzheimer’s Disease						
Cases of death, *n*	285	377	359	403		
Crude HR (95% CI)	1.00	1.00 (0.86–1.17)	1.01 (0.87–1.18)	1.15 (0.99–1.34)	0.020	1.01 (1.00–1.02)
Sex and Age-Adjusted HR (95% CI)	1.00	1.06 (0.90–1.23)	1.03 (0.88–1.21)	1.13 (0.97–1.32)	0.117	1.01 (1.00–1.02)
Multivariable-Adjusted HR (95% CI) ^2^	1.00	1.07 (0.90–1.27)	1.01 (0.85–1.20)	1.09 (0.92–1.29)	0.423	1.01 (0.99–1.02)
Chronic Liver Disease						
Cases of death, *n*	160	173	161	178		
Crude HR (95% CI)	1.00	0.82 (0.66–1.02)	0.81 (0.65–1.01)	0.90 (0.73–1.12)	0.858	1.01 (0.99–1.02)
Sex and Age-Adjusted HR (95% CI)	1.00	0.87 (0.70–1.08)	0.83 (0.67–1.04)	0.87 (0.70–1.08)	0.582	1.00 (0.98–1.02)
Multivariable-Adjusted HR (95% CI) ^2^	1.00	0.84 (0.66–1.06)	0.88 (0.69–1.13)	0.92 (0.72–1.18)	0.886	1.01(0.99–1.03)
Nephritis, Nephrotic Syndrome and Nephrosis						
Cases of death, *n*	165	176	172	202		
Crude HR (95% CI)	1.00	0.81 (0.65–1.00)	0.84 (0.68–1.04)	1.00 (0.81–1.22)	0.169	1.01 (0.99–1.02)
Sex and Age-Adjusted HR (95% CI)	1.00	0.87 (0.70–1.07)	0.86 (0.70–1.07)	0.96 (0.78–1.18)	0.586	1.00 (0.98–1.02)
Multivariable-Adjusted HR (95% CI) ^2^	1.00	0.83 (0.65–1.06)	0.89 (0.70–1.13)	0.88 (0.70–1.12)	0.799	0.99 (0.97–1.01)
Accident, Suicide or Homicide						
Cases of death, *n*	495	576	550	638		
Crude HR (95% CI)	1.00	0.88 (0.78–1.00)	0.89 (0.79–1.01)	1.05 (0.93–1.18)	0.014	1.01 (1.01–1.02)
Sex and Age-Adjusted HR (95% CI)	1.00	0.95 (0.84–1.07)	0.93 (0.82–1.05)	1.00 (0.89–1.13)	0.458	1.01 (1.00–1.02)
Multivariable-Adjusted HR (95% CI) ^2^	1.00	0.90 (0.79–1.02)	0.88 (0.77–1.00)	0.97 (0.85–1.10)	0.509	1.01(0.99–1.01)
Other/ Unknown Causes of Death						
Cases of death, *n*	1862	2260	2272	2485		
Crude HR (95% CI)	1.00	0.92 (0.87–0.98)	0.98 (0.92–1.04)	1.09 (1.02–1.15)	<0.001	1.01 (1.01–1.02)
Sex and Age-Adjusted HR (95% CI)	1.00	0.97 (0.92–1.03)	1.01 (0.95–1.07)	1.06 (0.99–1.21)	0.007	1.01 (1.00–1.01)
Multivariable-Adjusted HR (95% CI) ^2^	1.00	0.98 (0.91–1.05)	1.01 (0.95–1.09)	1.06 (0.99–1.13)	0.023	1.01(1.00–1.01)

^1^ Intake density is based on gram per 1000 kcal. C, Category. IQR, Inter Quartile Range; ^2^ Multivariable models were adjusted for age (years), sex (male or female), BMI (<25, ≥25 to <30, ≥30), level of education (1–11 years of schooling, 12 years/high school, high school/college, college/ postgraduate), race (Non-Hispanic White, Non-Hispanic Black, Other), self-reported health condition (excellent, very good, good, fair/ poor), smoking status (never, former: ≤20 cigarettes/d, former: >20 cigarettes/d, current: ≤20 cigarettes/d, current: >20 cigarettes/d), total energy consumption (in kilocalories per day), alcohol consumption (grams per day), vitamin consumption (yes or no), physical activity (never, rarely, 1–3 times/month, 1–2 times/week, 3–4 times/week, ≥5 times/week) and food groups including white meat (grams per day), red meat (grams per day), whole grain (grams per day), vegetable (grams per day) and fruit (grams per day). ^3^ Continuous for every 1.7 grams of increased peanut butter consumption.

**Table 4:** Hazard ratios for total mortality for those eating nuts/ peanut butter vs. those not eating nuts/ peanut butter and those eating nuts/ peanut butter only one to six times per year, stratified by subgroups of possible risk factors ^1^.

	Nut Intake (*n* = 374, 101)				Peanut Butter Intake (*n* = 380,351)			
	Number of Deaths	Number of Person-Years	HR (95% CI)	*p*-Value for Interaction	Number of Deaths	Number of Person-Years	HR (95% CI)	*p*-Value for Interaction
Sex				0.51				0.22
Female	163,068	2,405,610	0.86 (0.83–0.88)		166, 095	2,449,214	1.01 (0.98–1.04)	
Male	211,033	3,047,492	0.88 (0.86–0.90)		214, 256	3,092,462	1.02 (1.00–1.04)	
Age				0.86				0.25
<60	148,169	2,229,983	0.87 (0.84–0.91)		150, 276	2,261,425	1.01 (0.97–1.05)	
≥60	104,412	1,524,125	0.89 (0.86–0.92)		106, 093	1,547,996	1.00 (0.97–1.04)	
≥65	121,520	1,698,994	0.87 (0.85–0.89)		123, 982	1,732,253	1.02 (1.00–1.05)	
Education				0.29				0.13
1–11 years of schooling	91,071	1,309,986	0.87 (0.84–0.90)		92, 695	1,332,690	1.02 (0.99–1.06)	
12 years of schooling or completed high school	36,328	527,507	0.90 (0.85–0.95)		36, 739	533,243	1.00 (0.95–1.06)	
Post-high school or some college education	86,581	1,260,710	0.88 (0.85–0.91)		87, 713	1,276,987	1.02 (0.98–1.05)	
College and postgraduate education	150,552	2,218,676	0.86 (0.84–0.89)		152, 574	2,248,056	1.01 (0.98–1.04)	
Smoking Status				0.69				0.02
Never	140,903	2,102,481	0.89 (0.86–0.92)		143, 166	2,135,511	1.02 (0.98–1.05)	
Former	174,307	2,540,193	0.88 (0.86–0.90)		176, 654	2,573,900	1.03 (1.00–1.05)	
Current	45,194	614,019	0.88 (0.85–0.91)		45, 833	622,288	1.00 (0.96–1.04)	
BMI				0.11				0.04
<25	138,038	2,016,670	0.86 (0.83–0.88)		140, 290	2,048,781	1.04 (1.01–1.07)	
≥25	155,643	2,275,775	0.89 (0.86–0.91)		158, 016	2,309,579	1.05 (1.03–1.07)	
≥30	72,070	1,041,174	0.88 (0.84–0.91)		73, 226	1,057,435	1.02 (0.98–1.06)	
Health Condition				0.73				0.01
Excellent	79,625	1,188,674	0.86 (0.82–0.90)		80, 764	1,205,415	0.98 (0.94–1.03)	
Very Good	149,788	2,203,197	0.88 (0.85–0.91)		151, 843	2,233,073	1.00 (0.97–1.03)	
Good	116,648	1,677,765	0.87 (0.85–0.90)		118, 611	1,705,446	1.02 (0.99–1.05)	
Fair/Poor	22,891	311,061	0.88 (0.83–0.92)		23, 376	317,397	1.10 (1.04–1.16)	

NOTE: “One time” is not equivalent to “one serving.” ^1^ Multivariable models were adjusted for age (years), sex (male or female), BMI (<25, ≥25 to <30, ≥30), level of education (1–11 years of schooling, 12 years/high school, high school/college, college/ postgraduate), race (Non-Hispanic White, Non-Hispanic Black, Other), self-reported health condition (excellent, very good, good, fair/ poor), smoking status (never, former: ≤20 cigarettes/d, former: >20 cigarettes/d, current: ≤20 cigarettes/d, current: >20 cigarettes/d), total energy consumption (in kilocalories per day), alcohol consumption (grams per day), vitamin consumption (yes or no), physical activity (never, rarely, 1–3 times/month, 1–2 times/week, 3–4 times/week, ≥5 times/week) and food groups including white meat (grams per day), red meat (grams per day), whole grain (grams per day), vegetable (grams per day) and fruit (grams per day).
